# Genetic variation at 5 new autosomal short tandem repeat markers (D10S1248, D22S1045, D2S441, D1S1656, D12S391) in a population-based sample from Maghreb region

**DOI:** 10.3325/cmj.2011.52.368

**Published:** 2011-06

**Authors:** Venusia Cortellini, Nicoletta Cerri, Andrea Verzeletti

**Affiliations:** Department of Forensic Medicine, University of Brescia, Brescia, Italy

## Abstract

**Aim:**

To investigate allele distribution and genetic parameters of a population-based sample from Maghreb region.

**Methods:**

Allele frequencies for 5 new autosomal short tandem repeat (STR) markers (D10S1248, D22S1045, D2S441, D1S1656, and D12S391) and several forensic parameters were determined for 95 unrelated individuals.

**Results:**

The combined power of discrimination and power of exclusion for the 5 loci were high (0.9999991 and 0.9954757, respectively). Allele frequencies were compared with previously published population data. Significant differences were found between Maghreb population and all other populations at the locus D2S441. Also, significant differences were found between the Maghreb and the African American population at the D22S1045, D1S1656, and D12S391 loci, between Maghreb and Caucasian population at the D1S1656 locus, and between Maghreb and Hispanic population at the D22S1045 locus.

**Conclusions:**

Typing of the 5 new STR loci may provide a useful addition to the previously established sets of autosomal STRs.

Short tandem repeats (STR) are widely used for forensic testing. Ordinary paternity cases are solved by commercially available multiplexes kits, however, for more difficult cases, such as complex kinship analysis, additional STRs are needed to obtain better results. Besides, as many national DNA databases are growing and a large number of comparisons are being made within and between databases, concern for possible false-positive results may arise. This increases the need to introduce additional loci. The first European Standard Set (ESS) of loci included only 7 STRs loci, but the European Network of Forensic Science Institutes and the European DNA Profiling recommended to extend the ESS loci by adopting additional 3 miniSTRs loci (D10S1248, D22S1045, D2S441) and 2 additional polymorphic loci in 2006 (D1S1656, D12S391) (1,2).

These new 5 loci improve the discriminatory power of forensic analysis and, by amplifying fragments well below current average amplicon sizes, can enhance genotyping success when analyzing highly degraded DNA (3,4).

In order to verify and allow their use in forensics, the usefulness of ESS STR loci, it is necessary to obtain sufficient data from different populations.

## Methods

Saliva samples were obtained in 2010 from 95 unrelated, healthy immigrants from Maghreb region, whose both parents were born in Maghreb region (Morocco, Egypt, and Tunisia).

Genomic DNA was extracted from buccal swabs using Chelex^®^ 100 method (Biorad, Richmond, CA, USA) (5).

PCR amplifications were performed in a GeneAmp^®^ PCR System 9700 Gold Plate (Applied Biosystems, Foster City, CA, USA) using the commercial kit AmpFiSTR^®^ NGM (Applied Biosystems), according to manufacturer’s recommendations (6). Typing was performed by capillary electrophoresis using an ABI Prism^®^ 310 Genetic Analyzer (Applied Biosystems) and allele calling was performed with the software GeneMapperID V3.2 (Applied Biosystems), using manufacturer’s allelic ladders, bins, and panels.

For quality control, the laboratory regularly participates in the quality control proficiency testing programs provided by the GEDNAP group (German DNA Profiling, *http://www.gednap.org*).

Allele frequencies at each locus were calculated by direct counting. Statistical parameters of forensic interest were estimated: observed and expected heterozygosity (H*obs*, H*exp*) and standard error (7), polymorphism information content (8), power of discrimination (9), and power of exclusion (10). ARLEQUIN software, version 3.11 (11) was used to asses deviations from Hardy-Weinberg equilibrium. Allele frequencies were compared with previously published population data using an exact test through the ARLEQUIN software, version 3.11 (11).

## Results and discussion

A total of 95 samples were analyzed (Table 1). Deviation from Hardy-Weinberg equilibrium was detected for D22S1045 (*P* = 0.0037), D2S441 (*P* = 0.0006), and D12S391 (*P* = 0.0002) loci, even after a Bonferroni correction (12) for multiple testing (*P* < 0.0100). These deviations could be explained by an excess of homozygotes due to population substructure (Wahlund effect within the communities) or by a high inbreeding rate due to widespread endogamy (13,14). A larger sample size could help in understanding which of the two hypotheses is correct.

**Table 1:** Allele frequencies and statistical parameters for D10S1248, D22S1045, D2S441, D1S1656, and D12S391 short tandem repeat loci in a population sample from Maghreb region (n = 95)*

D10S1248	D22S1045	D2S441	D1S1656	D12S391	Allele
	0.0105				6
0.0053					8
0.0053		0.0053			9
		0.0894	0.0263		10
0.0105	0.1263	0.3632	0.0579		11
		0.0842			11.3
0.0632	0.0053	0.0632	0.1526		12
		0.0211			12.3
0.2526	0.0211	0.0053	0.1105		13
		0.0526			13.3
0.3263	0.1052	0.2789	0.0894		14
			0.0053		14.3
0.2367	0.3316	0.0263	0.1632	0.0263	15
			0.0474		15.3
0.0737	0.3368	0.0105	0.1421	0.0105	16
			0.0632		16.3
0.0211	0.0474		0.0421	0.1000	17
			0.0526	0.0158	17.3
	0.0158		0.0053	0.1947	18
			0.0316	0.0263	18.3
0.0053				0.1632	19
			0.0105	0.0263	19.3
				0.1368	20
				0.0053	20.3
				0.0789	21
				0.0737	22
				0.0684	23
				0.0474	24
				0.0211	25
				0.0053	26
0.7263	0.6000	0.6421	0.8737	0.7895	H*obs*
0.7636	0.7465	0.7672	0.8937	0.8852	H*exp*
4.3589 × 10^−2^	4.4631 × 10^−2^	4.3362 × 10^−2^	3.1624 × 10^−2^	3.27 × 10^−2^	SE
0.7365	0.0037	0.0006	0.0625	0.0002	HWE
0.7264	0.7076	0.7365	0.8843	0.8747	PIC
0.9084	0.8981	0.9029	0.9702	0.9660	PD
0.5470	0.5273	0.5702	0.7858	0.7705	PE

*Abbreviations: Hobs – observed heterozygosity; Hexp – expected heterozygosity; SE – standard error; HWE – *P* values from exact test for Hardy-Weinberg equilibrium; PIC – polymorphism information content; PD – power of discrimination; PE – power of exclusion.

The combined power of discrimination and power of exclusion for the 5 new ESS STR loci were 0.9999991 and 0.9954757, respectively. Based on heterozygosity, D1S1656 may be considered the most informative locus (H*obs* = 0.8737) and therefore especially useful in forensic investigations.

Allele frequencies for the 5 new ESS STR were compared with previously published population data (15-18) using an exact test and the ARLEQUIN software (11) (Table 2). No significant differences were found from the already published data for the locus D10S1248; significant differences were found between Maghreb population data and all other populations at the locus D2S441. Also, significant differences were detected between Maghreb and the African American population at the D22S1045, D1S1656, and D12S391 loci, as well as between Maghreb and Caucasian population at the D1S1656 locus, and between Maghreb and Hispanic population at the D22S1045 locus.

**Table 2:** Comparison of the allele frequencies for D10S1248, D22S1045, D2S441, D1S1656, and D12S391 loci between the Maghreb population and other populations*

Compared population	Exact test (*P* ± standard error)				
	D10S1248	D22S1045	D2S441	D1S1656	D12S391
Italians (Northern Italy) (15)	0.16620 ± 0.0278	0.09550 ± 0.0205	**0.00330 ± 0.0012**	0.71785 ± 0.0216	0.12015 ± 0.0242
Italians (Southern Italy) (16)	0.07545 ± 0.0198	0.31770 ± 0.0270	**0.01630 ± 0.0077**	0.93385 ± 0.0199	0.95185 ± 0.0107
Polish (17)	0.19565 ± 0.0392	0.51925 ± 0.0280	**0.01200 ± 0.0044**	0.22390 ± 0.0295	0.88705 ± 0.0164
African Americans (18)	0.06760 ± 0.0221	<**0.00001**	<**0.00001**	**0.02960 ± 0.0079**	**0.00020 ± 0.0002**
Caucasians (18)	0.11240 ± 0.0256	0.06825 ± 0.0147	**0.00025 ± 0.0003**	**0.01295 ± 0.0055**	0.35415 ± 0.0428
Hispanics (18)	0.17650 ± 0.0164	**0.02290 ± 0.0168**	<**0.00001**	0.16995 ± 0.0343	0.26690 ± 0.0342

*P – value of the exact test of population differentiation with 10 000 steps in the Markov chain length and 1000 steps of dememorization). In bold – significant differences (*P* < 0.05).

The obtained data demonstrate that these 5 new ESS STR loci are very useful for forensic purposes; the Maghreb population database could be helpful when testing individuals from this region.
